# Physical Activity Types Among School-Aged Children During COVID-19 Pandemic in Saudi Arabia: A Cross-Sectional Study

**DOI:** 10.3390/life15010091

**Published:** 2025-01-14

**Authors:** Mshari Alghadier, Reem M. Basuodan, Afnan Gmmash, Reem A. Albesher

**Affiliations:** 1 Department of Health and Rehabilitation Sciences, Prince Sattam Bin Abdulaziz University, Alkharj 11942, Saudi Arabia; m.alghadier@psau.edu.sa; 2 Department of Rehabilitation Sciences, Princess Nourah Bint Abdulrahman University, Riyadh 11671, Saudi Arabia; rmbasoudan@pnu.edu.sa; 3 Department of Physical Therapy, Faculty of Medical Rehabilitation Sciences, King Abdulaziz University, Jeddah 21589, Saudi Arabia; asgmmsh@kau.edu.sa

**Keywords:** physical activity, school age, children, COVID-19

## Abstract

Background: The COVID-19 pandemic may have had long-lasting detrimental effects on children’s physical health. Previous studies have shown that children’s participation in physical activity (PA) declined during the pandemic. This study examined the effect of the COVID-19 pandemic on PA type selection and the influence of gender, number of siblings, residence type, and caregiver education level on PA. Methods: Parents of Saudi children (ages 6–9 years) were recruited through convenience sampling and completed an online survey between July and August 2020. The parent-reported survey included demographics and PA types across three time periods (pre-, during, and post-lockdown). Chi-squared tests and logistic regression with pairwise comparisons were used to analyze the differences. Results: Parents reported that children (*n* = 361, mean age 7.7 ± 1.1 years) selected different PA types pre-COVID-19 pandemic more often than during the COVID-19 lockdown, such as swimming (16.9% vs. 12.8%), high-intensity jumping (9.8% vs. 6.6%), cycling (12.8% vs. 9.6%), football (14.3% vs. 6.1%), running (9.3% vs. 5.5%), virtual gaming exercise (5% vs. 3.2%), and playground activity (11.3% vs. 5.8%) (*p* < 0.05). Additionally, PA type was shown to be influenced by gender and residence type, with girls being 55% more likely to be physically active during COVID-19 compared to boys, and participants living in houses without private yards being less physically active compared to those who lived in houses with private yards. Conclusions: Children’s gender (boy vs. girl) and residence type (with private yards vs. without private yards) affected their PA level during the COVID-19 lockdown. These findings suggest that more effort should be directed toward understanding the influence of gender and house types in the selection of PA types.

## 1. Background

The Canadian 24 h movement guidelines have been developed to improve health outcomes in children and youths [1]. These guidelines state that children should be involved in at least 60 min of moderate-to-vigorous intensity physical activity (PA) daily, should not exceed two hours of sedentary recreational screen time daily, and should sleep for an uninterrupted 9–11 h per night [1]. Moderate intensity PA includes activities that allow a person to engage in a conversation comfortably while accelerating their breathing level and heart rate. On the other hand, activities in vigorous intensity PA elevates a person’s heart rate and breathing substantially, making it challenging for them to talk. A recent systematic review and meta-analysis including 387,437 participants from 23 countries showed that most children failed to meet these three 24 h movement guidelines and that levels of physical inactivity increased during the COVID-19 pandemic [2]. In Arab countries, the prevalence of inactivity among children was reportedly high using self-reported tools [3]. Based on self-reported measures, school-age children in Saudi Arabia are far from meeting the global guidelines, and the COVID-19 pandemic has had a negative impact on their daily PA and screen time [4].

Physical inactivity can be a fundamental cause of various public health problems, such as obesity, diabetes, high blood pressure, and even cancer [5,6,7]. The World Health Organization ranks physical inactivity as the fourth leading cause of global mortality, estimating that it results in 3.2 million deaths globally, mainly due to cardiovascular disease, diabetes, hypertension, and some cancers [5]. Physical inactivity in childhood heightens the likelihood of developing chronic diseases in adulthood. Children’s persistent sedentary behaviors increase their risk of adopting an ongoing inactivity pattern as they age. Promoting physical fitness early in life is critical to sustaining an active lifestyle and preventing adverse health outcomes [8,9].

Sibling dynamics can significantly influence children’s physical health. Having siblings provides children with numerous opportunities to be physically active. Children with multiple siblings have regular peer motivation and support to engage in sports, cooperative play, and competitive activities. Living with multiple siblings could also negatively affect some children if they have diverse interests, limited resources, or share sedentary activities. More studies should be conducted to understand the impact of the number of siblings on physical activity in stressful situations such as during a pandemic [10,11,12].

Other factors such as children’s residence type and their caregiver’s educational level should be considered when measuring children’s PA level. Differences in physical activity levels were previously reported in youths living in houses vs. flats. A study by Aguilar-Farias and colleagues found that children living in apartments were less active during the pandemic [13]. This finding must be further investigated to gain deeper insight into the relationship between residence type and physical activity in school aged children. The relationship between a caregiver’s education and their children’s PA level is controversial. Some studies suggest that children of highly educated caregivers engage in PA activities more than those of caregivers with lower educational qualifications. This could be attributed to the fact that caregivers with higher educational levels have a greater awareness of the health benefits associated with PA. However, some studies have shown that children of highly educated parents spend more time on screens and they are more sedentary in comparison to children of parents with lower levels of education. Thus, the relationship between caregiver education and children’s PA requires additional evidence [14,15].

Girls are less physically active than boys throughout childhood in all ethnic groups [2,3,6,7,16]. Klinker et al. showed that school-age girls are involved in fewer outdoor activities and spend less time on moderate-to-vigorous outdoor PA than boys [17]. Factors that may explain these gender differences include gender socialization and social norms [18]. In general, COVID-19 affected boys and girls differently, presenting higher fatality rates and worse prognoses in males [19] and higher rates of psychological impacts in females [20].

Physical activity is critical for school-aged children as it could determine their activity levels for the rest of their lives. Recognizing the changes that occur in children’s PA before and after a major disaster happens is essential to preventing any future detrimental consequences that could arise if a similar situation develops. The results of this study will be used to add evidence to support meeting children’s suggested levels of PA and determine predicting factors that could limit their participation in PA. The COVID-19 pandemic has had a negative impact on the physical activity levels of school-age children [4,21]. COVID-19 restriction disturbed access to schools, playgrounds, and structured sport venues. As a result of these factors, children have faced significant barriers to maintaining regular physical activity [22]. In general, COVID-19 has affected boys and girls differently, with higher fatality rates and worse prognoses in males [19] and higher rates of psychological impacts in females [20]. During COVID-19, Saudi Arabia implemented a nationwide lockdown and physical distancing on 23 March 2020. A 24 h lockdown was implemented by 2 April 2020 in four governorates and other Saudi cities as the number of confirmed cases rose. Although the lockdown ended in June 2020, social isolation, mask wearing, and school closures persisted until January 2022.

The aims of this study were as follows: (a) to examine the participation of school-age children in different types of PA across three time periods—before the COVID-19 pandemic, during the COVID-19 lockdown, and after the lockdown (the last 7 days during the pandemic when social distancing applied but there was no curfew); and (b) to explore the influence of gender, number of siblings, residence type, and caregiver education level on type of PA selection among school-age children. We hypothesized that school-age girls would have selected less vigorous PA types than boys and shown greater physical inactivity as a result of the pandemic.

## 2. Methods

A survey was conducted online between July and August 2020, after lockdown restrictions were gradually eased, among caregivers of 6- to 9-year-old children living in Saudi Arabia. The data were collected during the summer holidays. Several social media platforms (Twitter, WhatsApp, Instagram, and Snapchat) were used to promote the survey. Participants had to meet the following inclusion criteria: (i) caregiver of a child living in Saudi Arabia, (ii) caregiver of a 6- to 9-year-old child, and (iii) being capable of reading Arabic. To conduct the survey, authors RA and RB developed online questionnaires. In accordance with the Helsinki Declaration for Ethical Standards in Human Research, the Princess Nourah bint Abdulrahman University Ethics Committee approved study H-01-R-059 in Riyadh, Kingdom of Saudi Arabia. All methods were performed in accordance with relevant institutional review boards and regulations. The study protocol, procedures, and respondents’ rights were explained at the beginning of the study. All respondents provided informed consent before completing the survey.

### 2.1. Survey Components

During the parent-reported survey, demographic information and information on children’s PA types were collected across three time periods: pre-COVID-19 pandemic, during the lockdown during the COVID-19 pandemic, and the last 7 days during the COVID-19 pandemic where social distancing applied but there was no lockdown. Prior to the start of data collection, eight mothers from different regions of Saudi Arabia completed a pilot survey and reviewed it for clarity and functionality. Each user was allowed one attempt to fill out the survey for one child. The following sections were included in the survey:

1. Demographic information

This part of the questionnaire contained nine questions about each child’s age, gender, number of siblings, health status, area of residence, and type of residency, as well as the caregiver’s relationship with the child, place of residence, educational level, and employment during the lockdown. There are five main living areas in Saudi Arabia: the central region, western region, eastern region, southern region, and northern region. The central region is the most populated area, followed by the eastern region, and these factors might influence PA levels.

2. Physical activity types:

In this section, PA was first defined and caregivers were asked to provide information regarding the PA types that their children practiced across the three time periods. The response format for the PA questions consisted of multiple-choice questions. A PA types question was “Has your child practiced one or more of the following types of physical activity: swimming, high-intensity jumping, cycling, rope jumping, football, basketball, volleyball, high-intensity climbing, high-intensity running, walking, dancing, yoga, virtual gaming exercise, exercise with family member, playground activity, zumba?”. High-intensity jumping was defined as jumping in place with high intensity (such as using a trampoline), whereas rope jumping was defined as using a rope while jumping. The caregivers were asked to select one or more PA types. These PA questions are valid and reliable and were adopted and modified from previous research [23]. To ensure the validity of the Arabic version of the PA questions, a certified translation center translated the questions into Arabic in three steps using the same methodology [24]. The survey provided clear definitions of PA types.

### 2.2. Data Collection

Due to the rapid changes in the pandemic situation and restrictions, the online survey was distributed between 24 July 2020 and 5 August 2020 to ensure that the data were captured within a similar period. The data were collected during the period when local educational institutions were forced to close on 15 March 2020 and remained closed throughout the period of data collection.

### 2.3. Data Analysis

Statistical analyses were conducted using R version 4.0.3 (2020-10-10). Descriptive statistics were first analyzed to report frequencies and percentages for the categorical variables and to report the means and standard deviations (SDs) for the continuous variables. Prior to the data analysis, all variables were screened for normality. Chi-squared tests were used to test for statistically significant differences between the percentages of PA selection during the three time periods. To gauge gender differences in PA types, a chi-squared analysis was used to test the percentages of selection of the different PA types during the three time periods divided by gender (boy vs. girl). To examine the influence of different predictor variables on PA type selection, a logistic regression model was used. This was employed to analyze the relationship between age, gender, number of siblings, caregiver education level, and residence type and physical inactivity (no PA) during two periods (pre-COVID-19 and during the COVID-19 lockdown). A categorical test of the predictor variables was conducted by reducing each caregiver’s education level to two levels: intermediate, high school, and diploma into “not highly educated” and Bachelor’s, Master’s, and PhD into “highly educated”.

## 3. Results

A total of 361 participants met the inclusion criteria and were included in the study. Children’s ages ranged from 6 to 9 years, with an average of 7.66 years (SD = 1.1). Approximately 52.6% of the children were female, and the relative majority had at least one sibling (30.47%) and resided in Saudi Arabia’s central region (50.9%). Most caregivers had a bachelor’s degree or higher (83.64%), and more than 60% of all participants lived in houses with private yards. Table 1 summarizes the demographic characteristics of the participants.

The chi-squared test revealed significant differences in PA types (swimming, high-intensity jumping, cycling, football, running, walking, virtual gaming exercise, and playground activity) among the three periods studied. The majority of participants were not active during the COVID-19 lockdown (80), and activities such as swimming, high-intensity jumping, cycling, football, running, virtual gaming, and playground activities were more likely to be selected pre-COVID-19 compared to during and after the COVID-19 lockdown. The participants also showed significant differences in PA types (high-intensity jumping, cycling, football, running, walking, virtual gaming exercise, and playground activity) between pre-COVID-19 and after the COVID-19 lockdown. Table 2 summarizes the percentages of PA type selection across the three periods.

Table 3 shows descriptive statistics for the frequencies of children who selected different PA types during three time periods divided by gender. In the pre-COVID-19 period, girls were more likely to select rope jumping, dancing, yoga, virtual gaming exercise, exercise with a family member, playground activity, and Zumba. The data from COVID-19 lockdown period showed that girls selected rope jumping, volleyball, dancing, and Zumba more than boys. In the post-COVID-19 lockdown period, rope jumping, walking, dancing, yoga, exercise with a family member, and Zumba were more likely to be selected by girls. Overall, football was more likely to be selected by boys in all three time periods compared to girls.

Logistic regression was used to analyze the relationship between age, gender, number of siblings, caregiver education level, and residence type and physical inactivity (no physical activity) during two periods (pre-COVID-19 and during COVID-19 lockdown). The results of the logistic regression model showed that being a girl and having a higher number of siblings were predictive of a reduced risk of inactivity during the COVID-19 lockdown. The logistic regression model of the pre-COVID-19 period was not significant [X^2^ (6, n = 361) = 2.96, *p* = 0.81]. The model explained 2% (Nagelkerke’s R^2^) of the variance in physical inactivity during the pre-COVID-19 period. In contrast, the logistic regression of the during COVID-19 lockdown period was significant [X^2^ (6, n = 361) = 16.1, *p* = 0.01]. The model explained 6% (Nagelkerke’s R^2^) of the variance in physical inactivity during the COVID-19 lockdown period. The results showed a 45% reduction in being physically inactive in girls compared to boys, and participants living in houses without yards were twice as likely to be physically inactive than those living in houses with yards. Table 4 summarizes the logistic regression findings for physical inactivity during the pre-COVID-19 and during COVID-19 lockdown periods.

## 4. Discussion

The COVID-19 pandemic has negatively affected children’s overall health, particularly during strict social isolation and lockdowns [25]. This study examined parent-reported PA types in school-age children in Saudi Arabia across three periods: pre-COVID-19, during the lockdown, and post-lockdown. Parents reported a decline in their children’s PA from pre-COVID-19 to lockdown, including reduced activities such as swimming, cycling, high-intensity jumping, football, running, virtual gaming, and playground activities. However, family home activity levels increased during the lockdown period. Overall, this study supports previous findings that COVID-19 restrictions, such as school and sports center closures, negatively affected children’s PA participation and selection [25,26,27].

The most reported PA types pre-COVID-19 pandemic were social PA types (e.g., swimming, football, and playground activity). Their reduction during the lockdown is not surprising, given school and sport center closure/cancelation at this time, during which the majority of children spent their time inside the house, as reported by their parents. Similar findings were reported in the United States during the early COVID-19 pandemic, and it was found that the most common types of PA were unorganized play and unstructured activities, such as running, walking, and playing hide and seek and similar games [27]. The authors also reported that older children (9–13 years) showed a greater decrease in PA than younger ones (5–8 years), contrary to another study in the Czech Republic [26]. However, this was not supported by our findings because of the limited age group; therefore, further research is required to investigate the influence of age on PA types.

Previous studies have reported that girls are less active than boys and tend to choose different PA types [2,3,6,7,16]. Evidence indicated that many Saudi children did not meet the global PA recommendations [2], which increased their risk of serious health problems. This is especially the case for girls, who selected different PA types than their peer boys. School-aged girls are involved in fewer outdoor activities and spend less time on moderate-to-vigorous outdoor PA than boys [17]. A similar pattern of less activity variation was found in girls, who participated in fewer PA types than boys. High levels of inactivity have been observed in many countries in the region [3]. Factors that may further explain gender differences in PA include gender socialization and social norms [18]. Our findings are in line with earlier evidence indicating that COVID-19 affected the lives of boys and girls differently [19,20].

Perceptions of PA can also be changed through initiatives that provide information on the links between health and PA [17]. Furthermore, creating the right environment for activity can have a positive impact on the physical performance of both boys and girls [18]. However, it is important to take gender into account when designing PA initiatives and interventions [3,18]. Improving recreational facilities can be considered a useful strategy for improving overall PA levels, especially during a circumstance such as the COVID-19 pandemic. The development of new public parks as well as the renovation of existing ones and green spaces will make them more attractive to visitors and increase their overall use by both boys and girls [18]. There is a need for initiatives that provide information on the links between health and PA designed for girls, to encourage their engagement in sports [17]. A closer assessment of the effectiveness of these interventions may provide in-depth understanding and aid future efforts. Further, public policies that address challenges like school closures, physical distancing, and restricted access to organized sports or recreational facilities are necessary to promote higher physical activity levels in school-age children during pandemics and lockdowns. Public policies could include supporting gamified interventions that promote sustained activity levels [28], implementing virtual or hybrid physical education [29], and community-based interventions that engage families [30].

A major strength of our study is that we investigated the most common types of PA reported by parents and shed light on the differences in PA over three periods, including two levels of COVID-19 restrictions. Despite its strengths, this study has some limitations. As with most studies conducted early in the COVID-19 pandemic, the nature of online caregiver reports and the replication of collected data are the main limitations of this study. Additionally, because we recruited online, the trend in spontaneous responses to the sample may have had an impact. Another factor that could have affected the results during the previous 7 days is the extremely hot weather in Saudi Arabia from July to August, which may have influenced PA selection, especially in outdoor activities. Moreover, recruiting a different age group (older than the recruited sample) would strengthen our understanding of older children’s PA types and preferences. Parents, who are pivotal in shaping habits, exhibit various approaches based on their educational level. Children with highly educated parents tend to be more active [31]. A significant relationship between parental education and PA was also found in studies conducted during the pandemic [32]. In our study, most parents had at least a bachelor’s degree, which may have affected the results. To promote children’s PA levels, parents’ educational qualifications and activity levels must be considered when participation in PA is emphasized. In addition, teaching resilience is important to enable communities to navigate future unforeseen crises, such as pandemics. To increase the rates of PA during any lockdown period, educators could add practical sessions on PA to the curriculum, which must be practiced at home. Educators can also train students in techniques related to problem solving, adaptability, and stress management. Providing related digital resources that illustrate the importance of PA is critical for encouraging the implementation of home-based activities. In this case, if another crisis occurs, students and educators will be better prepared to handle the situation with the required resilience and creativity [33].

## 5. Conclusions

Our study contributes to a better understanding of the types of PA and the impact of gender on PA selection, and investigates whether COVID-19 has had a similar impact on both genders at school age. A major strength of our study is that we investigated the most common types of PA reported by parents and shed light on the differences in PA over three periods, including two levels of COVID-19 restrictions. Despite its strengths, this study has some limitations. As with most studies conducted early in the COVID-19 pandemic, the nature of online caregiver reports and the replication of collected data are the main limitations of this study. Additionally, because we recruited online, the trend in spontaneous responses to the sample may have had an impact. Another factor that could have affected the results during the previous 7 days is the extremely hot weather in Saudi Arabia from July to August, which may have influenced PA selection, especially in outdoor activities. Moreover, recruiting a different age group (older than the recruited sample) would strengthen our understanding of older children’s PA types and preferences. Parents, who are pivotal in shaping habits, exhibit various approaches based on their educational level. Children with highly educated parents tend to be more active [31]. A significant relationship between parental education and PA was also found in studies conducted during the pandemic [32]. In our study, most parents had at least a bachelor’s degree, which may have affected the results. To promote children’s PA levels, parents’ educational qualifications and activity levels must be considered when participation in PA is emphasized. In addition, teaching resilience is important to enable the community to navigate future unforeseen crises, such as pandemics. To increase the rates of PA during any lockdown period, educators could add practical sessions on PA to the curriculum, which must be practiced at home. Educators can also train students in techniques related to problem solving, adaptability, and stress management. Providing related digital resources that illustrate the importance of PA is critical for encouraging the implementation of home-based activities. In this case, if another crisis occurs, students and educators will be better prepared to handle the situation with the required resilience and creativity [33].

## Figures and Tables

**Table 1:** Description of child and caregiver characteristics.

Variables	Categories	Mean (SD)/n (%)
**Child demographic profile**		
Age	Years, mean (SD)	7.66 (1.1)
Sex	Female, n (%)	190 (52.63)
	Male, n (%)	171 (47.37)
Siblings	None, n (%)	29 (8.03)
	1, n (%)	110 (30.47)
	2, n (%)	104 (28.80)
	3, n (%)	57 (15.78)
	>3, n (%)	61 (16.89)
Area of living	Central region, n (%)	184 (50.96)
	Western region, n (%)	123 (34.07)
	Eastern region, n (%)	42 (11.63)
	Northern region, n (%)	6 (1.66)
	Southern region, n (%)	6 (1.66)
Residence type	House with yard, n (%)	232 (64.26)
	House without yard, n (%)	118 (32.68)
	Other, n (%)	11 (3.04)
**Caregiver demographic profile**		
Relationship to the child	Mother, n (%)	343 (95.01)
	Father, n (%)	11 (3.04)
	Other, n (%)	7 (1.93)
Highest level of education completed	Intermediate school, n (%)	4 (1.10)
	High school, n (%)	31 (8.58)
	Diploma, n (%)	24 (6.64)
	Bachelor’s, n (%)	218 (60.38)
	Master’s, n (%)	63 (14.40)
	PhD, n (%)	32 (8.86)

n, number; SD, standard deviation.

**Table 2:** Summary of the Chi-squared statistics for different physical activity types across three time periods.

Type of Physical Activity	Pre-COVID-19	During COVID-19 Lockdown	After COVID-19 Lockdown	X^2^	*p*
No physical activity	25 (2.3)	80 (7.4)	45 (4.2)	36	<0.001
Swimming	183 (16.9)	139 (12.8)	162 (15)	10.9	<0.01
Jumping (high intensity)	106 (9.8)	72 (6.6)	71 (6.6)	12.4	<0.01
Bicycle	139 (12.8)	104 (9.6)	74 (6.8)	28.3	<0.001
Rope jumping	40 (3.7)	27 (2.5)	28 (2.6)	3.62	0.16
Football	155 (14.3)	66 (6.1)	60 (5.5)	81.6	<0.001
Basketball	16 (1.5)	7 (0.6)	7 (0.6)	5.55	0.06
Volleyball	6 (0.6)	8 (0.7)	3 (0.3)	2.27	0.32
High intensity climbing	22 (2)	12 (1.1)	11 (1)	5.15	0.07
Running	101 (9.3)	60 (5.5)	60 (5.5)	19.1	<0.001
Walking	78 (7.2)	79 (7.3)	51 (4.7)	9.01	<0.05
Dancing	75 (6.9)	78 (7.2)	84 (7.8)	0.68	0.71
Yoga	5 (0.5)	8 (0.7)	8 (0.7)	0.87	0.64
Virtual gaming exercise	54 (5)	35 (3.2)	19 (1.8)	18.9	<0.001
Exercise with family member	52 (4.8)	63 (5.8)	49 (4.5)	2.35	0.31
Playground activity	122 (11.3)	44 (4.1)	74 (6.8)	49.7	<0.001
Zumba	23 (2.1)	18 (1.7)	22 (2)	0.70	0.70
I don’t know	1 (0.1)	1 (0.1)	8 (0.7)	9.89	<0.01
Other	72 (6.6)	38 (3.5)	78 (7.2)	18	<0.001

Data are presented as numbers (percentages).

**Table 3:** An analysis of descriptive statistics and chi-squared tests comparing the frequency of children selecting different types of physical activity in three different time periods: pre-COVID-19, COVID-19 lockdown, and after COVID-19 lockdown, divided by gender.

Type of Physical Activity	Pre-COVID-19			During COVID-19 Lockdown			After COVID-19 Lockdown		
	Male	Female	X^2^	Male	Female	X^2^	Male	Female	X^2^
No physical activity	14 (1.3)	11 (1)	0.80	46 (4.2)	34 (3.1)	4.22 *	27 (2.5)	18 (1.7)	3.29
Swimming	78 (7.2)	105 (9.7)	3.35	58 (5.4)	81 (7.5)	2.88	80 (7.4)	82 (7.6)	0.47
Jumping (high intensity)	46 (4.2)	60 (5.5)	0.74	31 (2.9)	41 (3.8)	0.67	35 (3.2)	36 (3.3)	0.13
Bicycle	70 (6.5)	69 (6.4)	0.81	54 (5)	50 (4.6)	1.21	40 (3.7)	34 (3.1)	1.66
Rope jumping	4 (0.4)	36 (3.3)	25.2 **	3 (0.3)	24 (2.2)	15.4 **	5 (0.5)	23 (2.1)	10.6 *
Football	117 (10.8)	38 (3.5)	86.1 **	48 (4.4)	18 (1.7)	20.8 **	48 (4.4)	12 (1.1)	30.7 **
Basketball	10 (0.9)	6 (0.6)	1.53	4 (0.4)	3 (0.3)	0.27	6 (0.6)	1 (0.1)	4.21 *
Volleyball	2 (0.2)	4 (0.4)	0.48	1 (0.1)	7 (0.6)	3.99 *	2 (0.2)	1 (0.1)	0.45
High intensity climbing	10 (0.9)	12 (1.1)	0.95	9 (0.8)	3 (0.3)	3.80	5 (0.5)	6 (0.6)	0.01
Running	48 (4.4)	53 (4.9)	0.001	24 (2.2)	36 (3.3)	1.56	30 (2.8)	30 (2.8)	0.19
Walking	32 (3)	46 (4.2)	1.61	33 (3)	46 (4.2)	1.27	17 (1.6)	34 (3.1)	4.96 *
Dancing	9 (0.8)	66 (6.1)	47.5 **	16 (1.5)	62 (5.7)	28.8 **	15 (1.4)	69 (6.4)	38.2 **
Yoga	0 (0)	5 (0.5)	4.56 *	2 (0.2)	6 (0.6)	1.64	1 (0.1)	7 (0.6)	3.99 *
Virtual gaming exercise	15 (1.4)	39 (3.6)	9.78 *	12 (1.1)	23 (2.1)	2.66	6 (0.6)	13 (1.2)	2.01
Exercise with family member	17 (1.6)	35 (3.2)	5.25 *	23 (2.1)	40 (3.7)	3.61	15 (1.4)	34 (3.1)	6.39 *
Playground activity	39 (3.6)	83 (7.7)	17.53 **	21 (1.9)	23 (2.1)	0.002	34 (3.1)	40 (3.7)	0.07
Zumba	2 (0.2)	21 (1.9)	14.74 **	4 (0.4)	14 (1.3)	4.81*	3 (0.3)	19 (1.8)	10.69 *
I don’t know	0 (0)	1 (0.1)	0.90	0 (0)	1 (0.1)	0.90	7 (0.6)	1 (0.1)	5.28 *
Other	36 (3.3)	36 (3.3)	0.25	22 (2)	16 (1.5)	1.88	39 (3.6)	39 (3.6)	0.27

*p* < 0.05 *, *p* < 0.001 **; Data are presented as numbers (percentages).

**Table 4:** Logistic regression of the predictor variables (age, gender, number of siblings, caregiver education level, and residence type) in two time periods (Pre-COVID-19 and during COVID-19 lockdown).

Predictors	Odds Ratio	95% CI	*p*
**A: pre-COVID-19 period**			
Age, years	1.08	0.73–1.61	0.68
Gender			
Male	Reference category		
Female	0.65	0.28–1.48	0.30
Number of siblings	1.23	0.86–1.76	0.23
Caregiver education level			
Highly educated	Reference category		
Not highly educated	0.87	0.28–2.73	0.82
Residence type			
House with yard	Reference category		
House without yard	1.42	0.58–3.47	0.43
Other	1.64	0.18–14.6	0.65
**B: During COVID-19 lockdown period**			
Age, years	0.95	0.75–1.21	0.73
Gender			
Male	Reference category		
Female	0.55	0.33–0.993	0.02
Number of siblings	0.93	0.74–1.17	0.56
Caregiver education level			
Highly educated	Reference category		
Not highly educated	0.83	0.40–1.74	0.63
Residence type			
House with yard	Reference category		
House without yard	2.06	1.19–3.54	0.009
Other	4.17	1.13–15.42	0.03

## Data Availability

The datasets used and/or analysed during the current study are available from the corresponding author on reasonable request.
